# Structural and functional insights into Mimivirus ORFans

**DOI:** 10.1186/1471-2164-8-115

**Published:** 2007-05-09

**Authors:** Harpreet Kaur Saini, Daniel Fischer

**Affiliations:** 1 Computer Science and Engineering Dept., 201 Bell Hall, University at Buffalo, Buffalo, NY 14260-2000, USA; 2 Bioinformatics/Dept. of Computer Science, Ben Gurion University, Beer-Sheva 84015, Israel

## Abstract

**Background:**

Mimivirus isolated from A. *polyphaga *is the largest virus discovered so far. It is unique among all the viruses in having genes related to translation, DNA repair and replication which bear close homology to eukaryotic genes. Nevertheless, only a small fraction of the proteins (33%) encoded in this genome has been assigned a function. Furthermore, a large fraction of the unassigned protein sequences bear no sequence similarity to proteins from other genomes. These sequences are referred to as ORFans. Because of their lack of sequence similarity to other proteins, they can not be assigned putative functions using standard sequence comparison methods. As part of our genome-wide computational efforts aimed at characterizing Mimivirus ORFans, we have applied fold-recognition methods to predict the structure of these ORFans and further functions were derived based on conservation of functionally important residues in sequence-template alignments.

**Results:**

Using fold recognition, we have identified highly confident computational 3D structural assignments for 21 Mimivirus ORFans. In addition, highly confident functional predictions for 6 of these ORFans were derived by analyzing the conservation of functional motifs between the predicted structures and proteins of known function. This analysis allowed us to classify these 6 previously unannotated ORFans into their specific protein families: carboxylesterase/thioesterase, metal-dependent deacetylase, P-loop kinases, 3-methyladenine DNA glycosylase, BTB domain and eukaryotic translation initiation factor eIF4E.

**Conclusion:**

Using stringent fold recognition criteria we have assigned three-dimensional structures for 21 of the ORFans encoded in the Mimivirus genome. Further, based on the 3D models and an analysis of the conservation of functionally important residues and motifs, we were able to derive functional attributes for 6 of the ORFans. Our computational identification of important functional sites in these ORFans can be the basis for a subsequent experimental verification of our predictions. Further computational and experimental studies are required to elucidate the 3D structures and functions of the remaining Mimivirus ORFans.

## Background

Viruses are obligate intracellular parasites which are responsible for many diseases in plants and animals. They differ from other microorganisms in their extreme dependence on the host cell. In 2004, the genome of Mimivirus [GenBank:AY653733], was sequenced [1]. Mimivirus is a nucleocytoplasmic large DNA virus (NCLDV), and is the largest known virus, both in particle size (>0.4 μm in diameter) and genome length (1.2-Mbp) [1]. Its genome size is larger than that of several bacteria and archea [1,2], is characterized by an extensive gene repertoire and the absence of pseudogenes [1,3], and contains a total of 911 predicted protein-coding genes [1]. Previously characterized NCLDVs share a set of 31 evolutionary conserved genes required for viral replication, transcription and virion biogenesis [4]. Of these 31 genes, Mimivirus possesses 26, indicating that it might have originated from a common ancestor [1,5]. At the same time, it lacks 5 core NCLDV genes and contains a number of other unique genes, which clearly distinguish it from other viruses [5]. Strikingly, it contains genes related to tRNA modification, translation, protein folding, DNA repair, amino acid and lipid metabolism, which exhibit homology to eukaryotic genes [1,5]. Mimivirus is assigned to its own lineage at the beginning of the Eukarya branch, distinct from the other three domains of life (Eukarya, Bacteria and Archaea) [1]. Notably, out of 911 coded Mimivirus proteins, only 298 have been assigned functions based on homology [1]. This represents only 33% of the total predicted genes, which is typically a very low percentage in comparison to that of newly sequenced prokaryotic genomes (70%) [6]. Furthermore, over 300 of the Mimivirus proteins show no sequence similarity to any other protein in the databases. Proteins with no detectable sequence similarity to other proteins are referred to as "ORFans" [7]. All these characteristics make Mimivirus a unique virus and emphasize the need to understand its biology.

Here we focus on attempting to assign three-dimensional (3D) structures and putative functions to Mimivirus ORFans. Recent studies [8,9] have also provided some hints regarding the function of a number of originally unannotated Mimivirus sequences. Our goal here is to use fold recognition (FR) methods to identify a number of very confident computational 3D structural assignments which we subsequently analyze in detail in order to arrive at specific functional predictions. For structural predictions we used FR methods available through the 3D-Jury Meta server [10]. Using very stringent criteria, we were able to assign 3D structures to 21 of the ORFans. By analyzing the predicted structures and the conservation of functional motifs, 4 ORFans (R843, L374, R277, L759) were predicted to be enzymes which we functionally characterized as carboxylesterase/thioesterase, metal-dependent deacetylase, P-loop kinase and 3-methyladenine DNA glycosylase, respectively. 2 ORFans (L834 and L529) were predicted to belong to the BTB/POZ domain family and the eukaryotic translation initiation factor 4e (eIF4e) family, respectively.

## Results

Highly confident structural predictions (INUB score > 12 and 3D-JURY score > 50; see Methods) were obtained for 21 Mimivirus ORFans. No annotation exists for any of these ORFans, which means that they are currently annotated as hypothetical proteins. These 21 structural predictions were analyzed in detail in order to identify those for which, in addition to the structural assignment, a confident functional prediction can be made.

### ORFans allowing confident structural and functional predictions

Analysis of the sequence-structure alignments, the annotations in various databases and the literature, allowed us to confidently predict the functions for 6 ORFans. Table 1 shows for each ORFan, the 3D-Jury score, the PDB template chosen and its fold type. The last two columns list the predicted function and the functional evidence that allowed us to arrive at the confident functional prediction. In what follows we describe in detail the functional predictions for 3 ORFans. The results of remaining 3 ORFans are available online [11].

### ORFan R843

The 10 highest scoring results obtained by 3D-JURY are listed in Table 2. For each result, the table shows the 3D-Jury score, the server from which the model was obtained, the template used in the sequence-structure alignment and its SCOP [12] family classification. The highest 3D-Jury score was 119.75, well above the confidence threshold of 50. Furthermore, all the top ten predictions had scores above 86.75, corresponding to templates belonging to the α/β hydrolase superfamily. The table shows that similar results were obtained by the various servers used by 3D-Jury. Taken together, there is a strong indication of a very confident structural prediction.

The α/β hydrolase superfamily is one of the largest groups of structurally related hydrolytic enzymes which are highly divergent on the sequence level and perform a wide range of catalytic functions [13-16]. The canonical α/β hydrolase fold is constructed of eight β-strands connected by five α-helices [13,14,17]. Members in this family include a large number of enzymes such as acetylcholinesterase, dienlactone hydrolase, lipase, thioesterase, serine carboxypeptidase, haloalkane dehalogenase, haloperoxidase, lyase and others. Despite the marked variance in the primary structures, it has been found that all of these enzymes contain a catalytic triad, which is conserved in the invariant order of nucleophile-acid-histidine (but except from the histidine, the amino acid identities can vary) [14-16]. Although the fold recognition (FR) results unambiguously suggest that ORFan R843 is a member of this fold, predicting the specific family within the α/β hydrolases is not straightforward [18].

The 8 top FR results identify templates belonging to a single SCOP family, carboxylesterase/thioesterase (Table 2). Carboxylesterase/thioesterases catalyze the hydrolysis of compounds containing the functional groups such as carboxylic acid ester, amides and thioesters [19]. They are characterized by a conserved HG sequence, which constitutes the oxyanion hole, located about 70–100 amino acids ahead of the active site serine residue [20] and more specifically, carboxylesterases are characterized by the PROSITE [21] signature pattern containing the active site serine, [LIV]-X- [LIVFY]- [LIVMST]-G- [HYWV]-**S**-X-G- [GSTAC] [22]. In addition, other characteristics of this family (shared also with other families in the fold) include a similar catalytic triad (Ser-His-Asp/Glu) responsible for the nucleophilic attack on the carbonyl carbon atom of the ester bonds and a conserved pentapeptide sequence around the nucleophile, Gly-X-Ser-X-Gly, which is usually located between a β-strand and an α-helix and assumes an extremely sharp turn called a nucleophilic elbow [14].

The first 3D-JURY hit corresponds to the template 1fj2A, human acyl protein thioesterase 1 (APT1). APT1 is a canonical α/β-hydrolase fold, with the catalytic site made up of Ser-114, Asp-169 and His-203 [23]. It lacks the first β-strand and a typical insertion after strand β6, but contains a long loop connecting strand β4 with helix αB [23].

**Figure 1:**
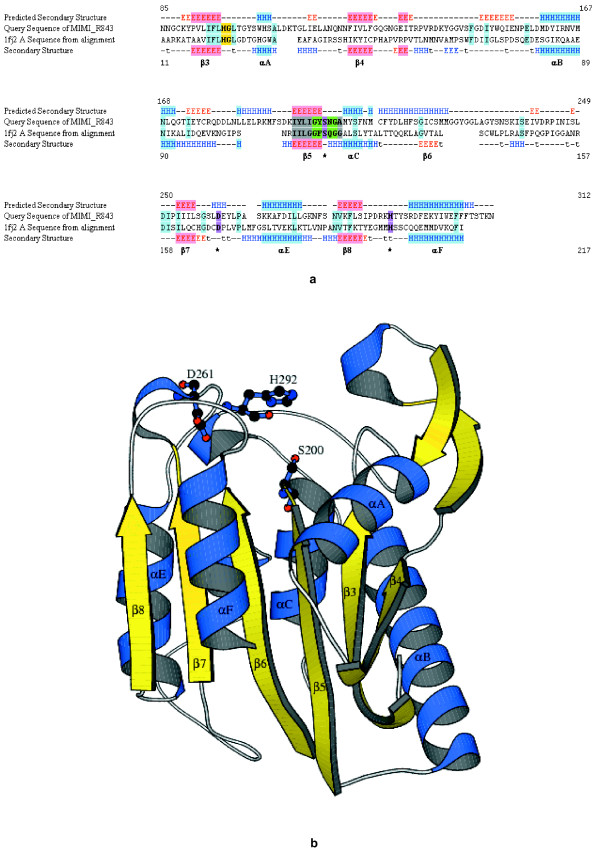
**a) **Sequence-structure alignment of the ORFan R843 and the template human acyl protein thioesterase 1 (PDB code: 1fj2, Chain: A). The predicted secondary structure (H: helix and E: β-strand) of R843 is shown above its sequence. The observed secondary structure of 1fj2A is shown below its sequence. Identical residues are marked in black with blue background. The secondary structural elements of 1fj2 are represented as α and β and are numbered from A-F and 3–8 respectively. The conserved catalytic residues, S, D and H are shown as stars and highlighted with bold black letters in magenta background. The conserved pentapeptide sequence G-X-S-X-G with active site serine is shown in green color. The conserved PROSITE signature pattern of carboxylesterases is shown in grey boxes. The residues probably forming the oxyanion hole are marked in bold black letters in yellow background. **b) **Ribbon diagram of predicted model of ORFan R843. The model was generated from INUB and refined using Nest [53]. α-helices and β-sheets are colored in blue and yellow. The remaining secondary structure is in white color. α-helices and β-strands of the canonical fold are numbered alphabetically from A to F and numerically from 3 to 8 respectively (the canonical strands 1 and 2 are missing). The residues forming the catalytic triad (Ser200, Asp261 and His292) are represented in ball-and-sticks. The figure is drawn using MOLSCRIPT program [54].

Figure 1a shows the sequence-structure alignment of R843 with the template 1fj2A generated by INUB. A ribbon diagram of the predicted three-dimensional (3D) model for R843 is shown in Figure 1b. The sequence identity between 1fj2A and R843 is only 15%. The aligned secondary structure of the template (Fig. 1a) is in good agreement with the predicted secondary structure of R843 (44% secondary structure match, with most of the secondary structure elements in the canonical fold predicted correctly). The loop between β4 and αB aligns to residues in R843, indicating that R843 is consistent with this feature of 1fj2A. The alignment revealed that the 'HG' motif (residues 97–98 in R843, yellow boxes in Fig. 1a), characteristic of caboxylesterase/thioesterase, is aligned to identical residues in R843. Interestingly, the PROSITE signature pattern containing the active site serine (shown in grey background), characteristic of carboxylesterases, is also conserved in R843. The catalytic residues, Ser, Asp and His (magenta boxes and marked by asterisks, Fig. 1a) are aligned to identical residues in R843. The assignment of the residues forming the catalytic triad is further corroborated by the fact that the active-site serine residue is a part of the conserved pentapeptide motif, G-Y-S-N-G (marked in green boxes, residues 198–202). Moreover, it is also evident from Figure 1b that R843 has a α/β hydrolase fold with all the secondary structure elements β3–β8 and αA-αF being conserved. It contains the active site residues in the same topological location with Ser located in a sharp turn connecting strand β5 and helix αC and Asp and His residues located after strands β7 and β8 respectively (Fig. 1b). In summary, based on the strong FR results and the conserved features in the sequence-structure alignment, we confidently predict that R843 is a member of the carboxylesterase/thioesterase family.

R843 is considered an ORFan sequence because BLAST [24] identified no similar sequences in the nonredundant (NR) database. However, searches in other databases confirm our result; CDD [25] and InterPro [26] indicated significant matches to the "abhydrolase_2" family which consists of both phospholipases and carboxylesterases. Similarly, COG [27] analysis indicated that residues 91 to 267 in R843 align to the COG "predicted esterase" (COG0400). Thus, these searches further corroborate our prediction that R843 is a member of the carboxylesterase/thioesterase family and has esterase activity.

### ORFan R277

The top 10 3D-Jury results for R277 are listed in Table 3. All the top 10 hits are confident structural predictions with 3D-Jury scores above 50. They correspond to P-loop kinases and are members of the P-loop containing nucleoside triphosphate hydrolases (P-loop NTPases) SCOP superfamily. However, the last column in the table shows that the templates belong to 3 different SCOP families (1yj5 not yet classified).

P-loop NTPases hydrolyze the β-γ phosphate bond of a bound nucleoside triphosphate in a Mg^2+ ^dependent reaction. Structurally, they adopt a three-layered α/β sandwich configuration that contains regularly recurring α-β units with the β-strands forming a central, mostly parallel β-sheet surrounded on both sides by α-helices [28]. At the sequence level, the P-loop is characterized by two strongly conserved sequence motifs, termed the Walker-A and Walker-B motifs [28,29]. The Walker-A motif (typically, Gx_4_GK [T/S], where x is any residue) encompasses the first strand and helix, and is involved in binding the triphosphate moiety of the substrate NTP [30]. The Walker B motif, composed of conserved aspartate (typically, hhhhD, where h is a hydrophobic residue), encompasses the third conserved strand and coordinates a Mg^2+ ^ion [29,31,32]. This motif is generally less conserved among the P-loop NTPases [33].

P-loop kinases, which are one of the types of P-loop NTPases are ubiquitous enzymes that transfer the γ phosphate of ATP to a wide range of substrates. The substrate of a kinase can be a small molecule, lipid, or protein. The P-loop kinases share the Walker-A and B motifs with the rest of the P-loop NTPases [28]. Further, P-loop kinases can be distinguished from other major groups of P-loop NTPases by the presence of a mostly helical structure between strands β4 and β5. Structurally, the P-loop is covered by a helical lid containing the conserved arginine motif Rx(2–3)R at the distal end of helix α5, where the second conserved arginine (in some cases, lysine) interacts with the γ phosphate of ATP [28]. Sequence comparison studies have shown that the position of the conserved arginine motif varies among different polynucleotide kinases [33].

3D-Jury first hit corresponds to a mammalian polynucleotide kinase (mPnk) (PDB code: 1yj5). mPnk belongs to a group of phosphotransferases with hydroxyl group as an acceptor and catalyzes the transfer of a phosphate from ATP to the 5' end of either DNA or RNA. mPnk consists of 3 domains: FHA domain, phosphate domain and kinase domain [33]. R277 matches with its kinase domain and the sequence identity of the alignment is 16%.

**Figure 2:**
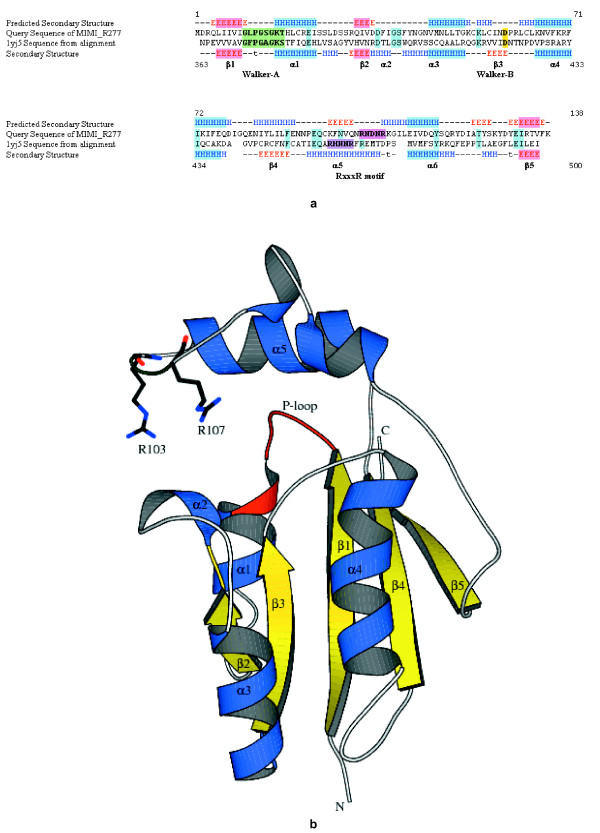
**a) **Sequence-structure alignment of ORFan R277 and the template mammalian polynucleotide kinase (PDB code: 1yj5, Chain: A). The ATP binding conserved motifs, Walker-A (P-loop) and Walker-B are highlighted in black with green and yellow backgrounds respectively. The conserved arginine motif in 1yj5 and R277 is shown in dark and light pink background respectively. **b) **Ribbon diagram of predicted model of ORFan R277. The model was generated from FFAS03 [55] and refined using Nest [53]. α-helices and β-strands are numbered numerically. The ATP-binding P-loop is highlighted in red color. The possible ATP interacting arginine residues (R103 and R107) present in the helical lid (helix α5) covering the P-loop are shown as sticks.

Figure 2a shows the sequence-structure alignment of R277 and 1yj5. Figure 2b shows the ribbon diagram of predicted 3D model of R277. The secondary structure match is 36%. Figure 2a reveals the conserved motifs, particularly the Walker-A and Walker-B motifs in R277, which are characteristic features of various P-loop NTPases. The walker-A motif, the P-loop sequence in R277 is ^10^GLPGSGKT^17 ^(marked in green color) and is identical to that of mPnk (^371^GFPGAGKS^378^). The walker-A motif is followed by the conserved walker-B (Asp-59) motif and is aligned well with that of the template (marked in yellow color). It is also clear from Figure 2b that P-loop (highlighted in red color) is located between the first β-strand and first α-helix, which is a common feature in many nucleotide dependent phosphotransferases. Structurally, in mPnk, the P-loop is covered by a helical lid which contains the conserved arginine motif, ^457^RHNNR^461^. Figure 2b shows a similar helical lid (helix α5 in Fig. 2b) folded over P-loop in R277. Further, the two conserved arginine residues in the helix lid probably are Arg-103 and Arg-107 in the sequence motif ^103^RNDNR^107 ^(shown as sticks at the distal end of α5 in Fig. 2b). The alignment in Figure 2a shows that the motif ^103^RNDNR^107 ^in R277 (shown in light pink) is not fully aligned and is six residues away from the motif ^457^RHNNR^461 ^of the template (shown in dark pink). Nevertheless, the presence of two arginines at distal end of helix α5 in R277 with the same orientation as that of the template is a strong indication that they may form the conserved arginine motif and interact with the bound ATP.

Based on the FR results, it can be inferred that R277 is a P-loop kinase with all the important sequence motifs conserved. To attempt to arrive to a more specific functional prediction, we also compared the Enzyme Commission (EC) numbers [34] (column 6 in Table 3) of the templates. Table 3 shows that the first 3 digits in the EC numbers are the same (EC 2.7.1.-), corresponding to phosphotransferases enzymes that transfer the phosphoryl group to the hydroxyl moiety. The last digit of an EC number usually represents the substrate specificity of a reaction, while the first three digits of the EC number usually describe the overall type of enzymatic reaction. Thus, based on the convincing features, we conclude that R277 is functionally related to the group of phosphotransferases that catalyzes a reaction involving the transfer of phosphoryl, where the substrate may be an alcohol moiety.

Confirming evidence was obtained from COG and Interpro (but not CDD), which also identified R277 as a member of the superfamily of P-loop containing nucleoside triphosphate hydrolases. In summary, we predict that R277 has a P-loop NTPase fold with conserved binding sites for a phosphate donor and may have a similar catalytic mechanism as that of other P-loop kinases.

### ORFan L529

For L529, the top 9 results obtained from 3D-Jury scored above 40, with the first 4 above 50 (Table 4). The first 9 hits correspond to the same SCOP family, the eukaryotic translation initiation factor 4E (eIF4E). The 10^th ^hit corresponded to an unrelated template with a low 3D-Jury score of 18.57.

**Figure 3:**
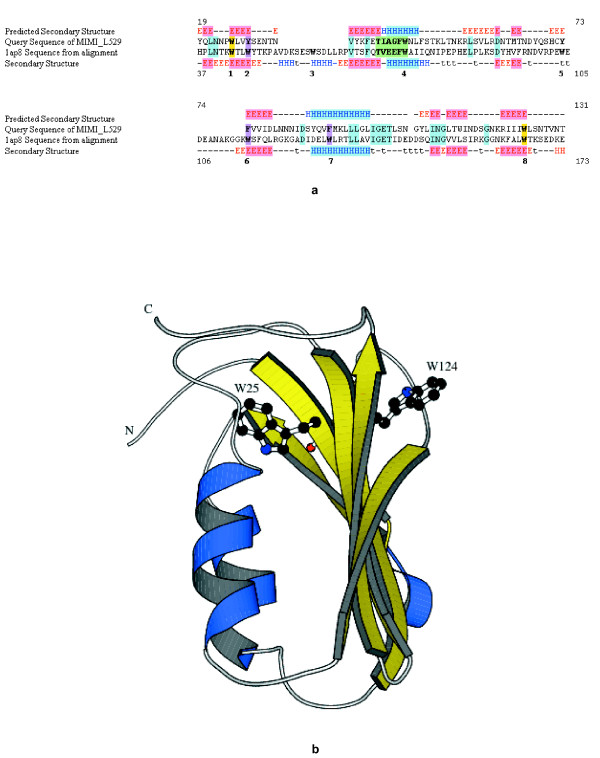
**a) **Sequence-structure alignment ORFan L529 and yeast translation initiation factor (PDB code: 1ap8). The eight conserved tryptophans of 1ap8 are numbered as 1–8. The conserved terminal tryptophans (Trp-1 & Trp-8) are shown in yellow boxes. The consensus motif (S/T)VxxxFW is highlighted in green color. **b) **Ribbon diagram of predicted model of ORFan L529. The model was built from FFAS03 [55] and refined using Nest [53]. The model shows curved antiparallel β-sheet with two long helices present on one side. The conserved tryptophan residues (Trp25 and Trp124) required for cap recognition are shown in ball-and-sticks.

eIF4E recognizes and binds the 7-methylguanosine-containing (m7Gppp) cap of eukaryotic cellular mRNAs during an early step in the initiation of protein synthesis and facilitates ribosome binding to mRNA [35]. The structure of eIF4E has been highly conserved throughout eukaryotic evolution and consists of eight antiparallel β-strands, 3 long and 3 short α-helices, whereby the 3 long helices are on one side of the β-sheet [36]. The cap analogue binds in a narrow slot on the concave surface [37-39]. Sequence analysis of eIF4E from several species revealed that all known eIF4Es contain a set of eight conserved tryptophans, two of which are critical for cap recognition and are absolutely conserved [40-42]. Site directed mutagenesis studies have shown that tryptophans 1 and 8 are essential for the cap recognition and the mutations of these two residues totally abolished the cap recognition, whereas other mutations had smaller or no effect on activity [41]. Recognition is mediated by π-π stacking between the 7-methyl-guanine and the indole groups of these two absolutely conserved tryptophan residues [37]. Moreover, eIF4E contains a phylogenetically conserved sequence (S/T)VxxFW, required for the interactions with eIF4Gs and 4E-BPs [36]. Substitution of Trp to a nonaromatic amino acid in this consensus sequence has been shown to disrupt the ability of eIF4E to interact with either eIF4G or with 4E-BPs [43,44].The highest scoring 3D-Jury corresponds to a translation initiation factor from yeast (PDB code: 1ap8). Figure 3a shows the sequence structure alignment of L529 with 1ap8. Figure 3b shows the ribbon diagram of our predicted 3D model for L529. The sequence and the secondary structure identities were 20% and 43%, respectively. Figure 3a reveals that L529 possesses tryptophan residues at positions equivalent to Trp-43, Trp-75 and Trp-166 of 1ap8. More significantly, tryptophans 1 (Trp-25) and 8 (Trp-124), important for cap recognition, are fully conserved as shown by yellow boxes. The consensus sequence (S/T)VxxFW (residues 39–44 of L529, marked in green color), including the Trp residue required for interaction with eIF4Gs and 4E-BPs is fully conserved, with the exception of an Ile residue in place of Val. Finally, L529 contains other aromatic residues (^28^Y, ^73^Y, ^74^F and ^89^F) at positions equivalent to other Trp resides (^46^W, ^104^W, ^115^W and ^130^W) in 1ap8 (marked in magenta background).

For this ORFan, no corroborating additional information was obtained from CDD, InterPro nor COG. Nevertheless, based on the absolute conservation of residues important for cap binding and the strong FR results, we predict that L529 is a translation initiation factor and hypothesize that it may participate in cap-dependent translation.

### ORFans allowing only confident structural predictions

For 10 of the 21 confident 3D-Jury predictions, we were able to predict their general fold only, but did not find strong evidence to attempt to arrive to a specific functional characterization. The 10 prediction results are summarized in Table 5. The table lists the ORFan name, the 3D-Jury score, the PDB template used and the predicted SCOP fold type. For each ORFan, the hits are confident fold assignments with 3D-Jury scores more than 50. Further, it was found that for 8 ORFans, the top confident (3D-Jury scores > 50) results belonged to the same SCOP family, however the key residues crucial for function and other family specific sequence motifs were not conserved. So, no clear functional annotations were obtained. Moreover, no CDD hits were obtained for any of the 10 ORFans. Searches against Interpro found 4 ORFans to contain known domains and were consistent with our FR results (see [11]).

### Ankyrins Prediction

5 ORFans (L146, R551, L677, R747 and R868) were predicted to be ankyrin repeats, with corresponding 3D-Jury templates 1n11, 1k1a, 1n0q, 1n0r and 1k3z. All 3D-Jury scores were above 50. 3D-Jury scores and CDD/InterPro searches of these ORFans are available online [11]. The ankyrin repeats are usually 33 amino acids long and are important for protein-protein interactions. Also, earlier studies of the Mimivirus genome have shown that Ankyrin repeats form the largest paralogous gene family [5,8] and are identified in more than 30 distinct ORFs [1].

## Discussion

Mimivirus is the largest DNA virus ever characterized. In terms of the size, complexity and gene repertoire, it has challenged the conventional views about viruses and thus is an interesting organism to study. In Mimivirus, only a small percentage of proteins have known functions derived from sequence homology. A large fraction of proteins have no homologs in current databases and constitutes a set of ORFans. Nothing is known about their structure and function. Despite their lack of homology to any other sequence in the databases, a fraction of the ORFans may correspond to very divergent members of known families. Using fold recognition we identified highly confident distant relationships to known proteins for 21 of the ORFans. Further, based on the 3D models and an analysis of the conservation of functionally important residues and motifs, we were able to derive functional attributes for 6 of the ORFans. In all these cases, the functionally important residues and sequence motifs were found to be fully conserved with respect to the template. 4 ORFans (R843, R277, L759 and L374) were predicted to be enzymes and we functionally categorized them as carboxylesterase/thioesterase, P-loop kinase, 3-methyladenine DNA glycosylase and metal-dependent deacetylase, respectively. Some of these enzymatic functions were also assigned to other Mimivirus ORFs by earlier studies [3].

ORFans R843 and R277 predicted as thioesterase and kinase respectively may play a role in cell regulation processes. Also, previous analyses have shown the presence of several types of DNA repair enzymes in Mimivirus including formamidopyrimidine-DNA glycosylases, UV-damage endonuclease and MutS protein and the presence of DNA repair enzymes is the one of the remarkable features of the Mimivirus genome [1,5]. ORFan L759 predicted as 3-methyladenine DNA glycosylase suggests that the ORFan correspond to a DNA repair enzyme which may function primarily by removing alkylation damage from duplex and single-stranded DNA. ORFan L374 predicted as deacetylase may play a role in lipid biosynthesis. 2 ORFans (L834 and L529) were predicted to be members of the BTB/POZ domain and eukaryotic translation initiation factor 4e (eIF4e) families, respectively. The BTB domain has also been found in some other ORFs (which were annotated as "unknown", including L834), making this family the second largest annotated paralogous family in Mimivirus [8]. BTB/POZ domains from several zinc finger proteins and have been shown to mediate transcriptional repression and to interact with components of histone deacetylase corepressor complexes [8]. ORFan L529 predicted as translation initiation factor 4e is an important protein of the translation apparatus and will function as a cap binding protein during protein synthesis and facilitate ribosome binding to mRNA.

We were unable to confidently assign specific functions to 10 of the confident structural predictions. In these cases, the fold assignment was fairly straightforward, but the further classification into precise protein family was not that evident. For example, the fold type for the ORFan R882 was predicted to be a 7-bladed β-propeller, but it was not possible to deduce any function since proteins with a β-propeller fold are involved in a wide range of biological functions, despite their structural similarity [45]. Further, it might be possible that these ORFans may exhibit some unrelated function while their 3D structures have converged to a similar fold. Finally, 5 ORFans were predicted to be ankyrin repeats. Ankyrin repeat is the most frequently found fold in Mimivirus and forms the largest paralogous family [8]. Ankyrin repeat-containing proteins are ubiquitously found in both viral and bacterial genomes and play structural roles.

Further computational and experimental work is needed to continue to unravel the mystery of the functions and origins of the many ORFans in Mimivirus. Until then, our knowledge about viral gene function will be limited, but progress is likely to be expected soon. In particular, since viruses are most abundant organisms in natural waters [46], Metagenomics projects [47,48] can further help in finding homologs. Indeed, in a recent study, an exhaustive similarity search of all Mimivirus predicted proteins against all publicly available sequences identified many of their closest homologues among the Sargasso Sea environmental sequences [9]. With the renewed interest in sequencing the vast viral repertoire, we will be able to unravel the functions and origins of more Mimivirus ORFans. Only experimental characterization, possibly guided by computational predictions, will allow a better characterization of the Mimivirus and other genomes.

## Conclusion

The present study describes the confident structural predictions for 21 of the ORFans in the Mimivirus genome using fold recognition. Based on the predicted 3D models and an analysis of the conservation of functionally important residues and motifs, we were able to derive functions for 6 of these ORFans. Indeed, the computational predictions can provide the basis for the subsequent experimental validation to unambiguously determine the exact functional roles of these ORFans.

## Methods

### Structure Prediction

Structure prediction was carried out using 3D-Jury [10], a fully automated protein structure meta prediction system that implements a number of fold recognition servers. The Meta-DP server [49] was used to identify possible domains within the protein sequences. Highly confident FR predictions are considered for INUB [50,51] and 3D-Jury scores greater than 12 and 50, respectively. After thousands of predictions processed by these servers, virtually no false positives have been observed above these thresholds.

### Motif Search

To confirm the 3D-Jury results and to obtain additional functional hints by other methods, we also searched for motifs (signature sequences) using the CDD [25], COG [27] and InterPro [26] databases.

### Function Prediction

Function assignment was carried out based on the conservation of features in the 3D-Jury sequence-template alignment, such as correspondence of predicted and observed secondary structure and conservation of functionally important residues (e.g. residues involved in catalytic activity, in binding to ligands, DNA or other proteins). To aid in the analysis, we developed a new automated Fold Recognition Alignment Analyzer tool called Fralanyzer [52]. Fralanyzer receives as input a sequence-template alignment from FR servers, automatically searches annotated databases (such as PDBSum and SwissProt) and highlights the functionally important positions that are identical in the alignment. Our predictions of functionally important residues as well as the annotated alignments in the figures were carried out with the aid of Fralanyzer.

## Authors' contributions

HKS conducted the computation, analyzed data, and drafted the manuscript. DF designed the research, supervised this project and finalized the manuscript. All authors read and approve the final manuscript.

## Figures and Tables

**Table 1:** Highly confident 3D structural and functional predictions for 6 Mimivirus ORFans.

**ORFan**	**3D-Jury score**	**3D-Jury First Hit**		**Predicted Fold**	**Predicted Function**	**Function specific Sequence Features**
		**PDB code**	**Name**			
**R843**	119.8	1fj2A	Human acyl protein Thioesterase 1	α/β-Hydrolase	Carboxylesterase/Thioesterase	Catalytic triad, 'HG' oxyanion motif and PROSITE signature pattern of carboxylesterase conserved.
**L374**	108.4	1q7tA	Metal-Dependent Deacetylase	LmbE-like	Metal-Dependent Deacetylase	Deacetyl 'AHPDDE' sequence motif and metal binding residues conserved.
**L834**	82.1	1r28A	B-cell Lymphoma 6 (bcl6) BTB domain	POZ domain	BTB domain	Residues Asp and Arg/Lys in the charged pocket of BTB domain, important for transcriptional repression conserved.
**R277**	63.9	1yj5A	Mammalian Polynucleotide Kinase	P-loop containing nucleoside triphosphate hydrolases	P-loop kinases	P-loop (walker-A and walker-B motifs) and arginine motif conserved.
**L759**	70.7	1ewnA	Human AAG DNA repair Glycosylase	FMT C-terminal domain-like	3-methyladenine DNA glycosylase	Active site residues Glu, Tyr, His, Tyr and Arg conserved.
**L529**	62.1	1ap8	Translation Initiation factor eIF4e	Translation Initiation factor eIF4e	Translation Initiation factor eIF4e	Trp residues important for CAP binding conserved.

**Table 2:** Fold recognition results for ORFan R843.

**Hit**	**3D-jury score**	**Server Name**	**PDB code**	**Protein Name**	**SCOP Family**
1	119.75	INUB_01	1fj2A	Human Acyl Protein Thioesterase 1	Carboxylesterase/thioesterase 1
2	113.75	FFA3_01	1fj2A	Human Acyl Protein Thioesterase 1	Carboxylesterase/thioesterase 1
3	110.75	INUB_03	1auoA	Carboxylesterase	Carboxylesterase/thioesterase 1
4	108.25	3DPS_01	1fj2A	Human Acyl Protein Thioesterase 1	Carboxylesterase/thioesterase 1
5	107.88	BasD_01	1fj2A	Human Acyl Protein Thioesterase 1	Carboxylesterase/thioesterase 1
6	105.25	3DPS_02	1auoA	Carboxylesterase	Carboxylesterase/thioesterase 1
7	100.38	ORF2_03	1fj2A	Human Acyl Protein Thioesterase 1	Carboxylesterase/thioesterase 1
8	99.12	FUG2_02	1auoA	Carboxylesterase	Carboxylesterase/thioesterase 1
9	86.88	FFA3_02	1gkkA	Feruloyl Esterase Domain	Carboxylesterase
10	86.75	FFA3_05	1gklA	s954a Mutant of the Feruloyl Esterase	Carboxylesterase

**Table 3:** Fold recognition results for ORFan R277.

**Hit**	**3D-jury score**	**Server Name**	**PDB code**	**Protein Name**	**EC number**	**SCOP Family**
1	63.88	FFA3_01	1yj5A	Polynucleotide kinase	2.7.1.78	-
2	59.00	BasD_01	1yj5A	Polynucleotide kinase	2.7.1.78	-
3	58.25	FUG2_01	1yj5A	Polynucleotide kinase	2.7.1.78	-
4	54.75	FFA3_03	1ly1A	T4 polynucleotide kinase	2.7.1.78	Nucleotide and Nucleoside Kinases
5	54.38	INUB_01	1ly1A	T4 polynucleotide kinase	2.7.1.78	Nucleotide and Nucleoside Kinases
6	53.50	FUG2_09	1shkA	Shikimate kinase	2.7.1.71	Shikimate kinase
7	52.62	FFA3_02	1qhsA	Chloramphenicol phosphotransferase	2.7.1.-	Chloramphenicol phosphotransferase
8	52.62	3DPS_01	1ly1A	T4 polynucleotide kinase	2.7.1.78	Nucleotide and Nucleoside Kinases
9	52.38	FUG2_06	1shkA	Shikimate kinase	2.7.1.71	Shikimate kinase
10	51.75	FFA3_06	1qhnA	Chloramphenicol phosphotransferase	2.7.1.-	Chloramphenicol phosphotransferase

**Table 4:** Fold recognition results for ORFan L529.

**Hit**	**3D-jury score**	**Server Name**	**PDB code**	**Protein Name**	**SCOP Family**
1	62.14	FFA3_03	1ap8	Translation Initiation Factor eIF4E	Translation Initiation Factor eIF4E
2	61.14	FFA3_02	1r58A	Translation Initiation Factor eIF4E in complex with m7gdp and eif4gi	Translation Initiation Factor eIF4E
3	52.14	3DPS_02	1ap8	Translation Initiation Factor eIF4E	Translation Initiation Factor eIF4E
4	51.57	FFA3_01	1ipbA	eIF4E complexed with 7-methyl gpppa	Translation Initiation Factor eIF4E
5	49.71	INUB_02	1ej1A	eIF4E bound to 7-methyl-gdp	Translation Initiation Factor eIF4E
6	48.57	BasD_01	1ap8	Translation Initiation Factor eIF4E	Translation Initiation Factor eIF4E
7	47.14	INUB_01	1ap8	Translation Initiation Factor eIF4E	Translation Initiation Factor eIF4E
8	44.29	ORF2_01	1ap8	Translation Initiation Factor eIF4E	Translation Initiation Factor eIF4E
9	41.86	3DPS_01	1ej1A	eIF4E bound to 7-methyl-gdp	Translation Initiation Factor eIF4E

**Table 5:** Highly confident 3D structural predictions for 10 Mimivirus ORFans.

**ORFan**	**3D-Jury score**	**Best Template**		**Fold Type**
		**PDB code**	**Name**	
**R882**	184.8	1gxr	WD40 region of human groucho/tle1	7-bladed beta propeller
**L201**	96.1	1s78	Receptor protein-tyrosine kinase erbb-2	Leucine rich repeat
**L237**	84.3	1c3j	Glucosyltransferase	UDP-Glycosyltransferase/glycogen phosphorylase
**R342**	67.5	1qpe	lymphocyte-specific kinase	Protein kinase like
**R467**	67.3	1gh2	Catalytic Domain of A New Human Thioredoxin-Like Protein	Thioredoxin fold
**L778**	60.6	1m38	Inorganic Pyrophosphatase	OB-fold
**R623**	58.8	1gr3	Crystal structure of a collagen VIII	TNF-like
**L649**	55.6	1r0w	Cystic fibrosis transmembrane conductance regulator nucleotide-binding domain	P-loop containing nucleoside triphosphate hydrolases
**L68**	53.9	1ji6	Insecticidal Bacterial Del Endotoxin Cry3Bb1	Galactose-binding domain-like
**R809**	52.7	1ciy	Insecticidal Toxin	Galactose-binding domain-like

## References

[B1] Raoult D, Audic S, Robert C, Abergel C, Renesto P, Ogata H, La Scola B, Suzan M, Claverie JM (2004). The 1.2-megabase genome sequence of Mimivirus. Science.

[B2] Koonin EV (2005). Virology: Gulliver among the Lilliputians. Curr Biol.

[B3] Claverie JM, Ogata H, Audic S, Abergel C, Suhre K, Fournier PE (2006). Mimivirus and the emerging concept of "giant" virus. Virus Res.

[B4] Iyer LM, Aravind L, Koonin EV (2001). Common origin of four diverse families of large eukaryotic DNA viruses. J Virol.

[B5] Suzan-Monti M, La Scola B, Raoult D (2005). Genomic and evolutionary aspects of Mimivirus. Virus Res.

[B6] Galperin MY, Koonin EV (2004). 'Conserved hypothetical' proteins: prioritization of targets for experimental study. Nucleic Acids Res.

[B7] Fischer D, Eisenberg D (1999). Finding families for genomic ORFans. Bioinformatics.

[B8] Suhre K (2005). Gene and genome duplication in Acanthamoeba polyphaga Mimivirus. J Virol.

[B9] Ghedin E, Claverie JM (2005). Mimivirus relatives in the Sargasso sea. Virol J.

[B10] Ginalski K, Elofsson A, Fischer D, Rychlewski L (2003). 3D-Jury: a simple approach to improve protein structure predictions. Bioinformatics.

[B11] Online data. http://fischerlab.cse.buffalo.edu/~hkaur/mimivirus/supple-info/.

[B12] Murzin AG, Brenner SE, Hubbard T, Chothia C (1995). SCOP: a structural classification of proteins database for the investigation of sequences and structures. J Mol Biol.

[B13] Ollis DL, Cheah E, Cygler M, Dijkstra B, Frolow F, Franken SM, Harel M, Remington SJ, Silman I, Schrag J (1992). The alpha/beta hydrolase fold. Protein Eng.

[B14] Heikinheimo P, Goldman A, Jeffries C, Ollis DL (1999). Of barn owls and bankers: a lush variety of alpha/beta hydrolases. Structure Fold Des.

[B15] Nardini M, Dijkstra BW (1999). Alpha/beta hydrolase fold enzymes: the family keeps growing. Curr Opin Struct Biol.

[B16] Holmquist M (2000). Alpha/Beta-hydrolase fold enzymes: structures, functions and mechanisms. Curr Protein Pept Sci.

[B17] Hotelier T, Renault L, Cousin X, Negre V, Marchot P, Chatonnet A (2004). ESTHER, the database of the alpha/beta-hydrolase fold superfamily of proteins. Nucleic Acids Res.

[B18] Siew N, Saini HK, Fischer D (2005). A putative novel alpha/beta hydrolase ORFan family in Bacillus. FEBS Lett.

[B19] Satoh T, Hosokawa M (1998). The mammalian carboxylesterases: from molecules to functions. Annu Rev Pharmacol Toxicol.

[B20] Satoh T, Hosokawa M (1995). Molecular aspects of carboxylesterase isoforms in comparison with other esterases. Toxicol Lett.

[B21] Falquet L, Pagni M, Bucher P, Hulo N, Sigrist CJ, Hofmann K, Bairoch A (2002). The PROSITE database, its status in 2002. Nucleic Acids Res.

[B22] Cousin X, Hotelier T, Giles K, Lievin P, Toutant JP, Chatonnet A (1997). The alpha/beta fold family of proteins database and the cholinesterase gene server ESTHER. Nucleic Acids Res.

[B23] Devedjiev Y, Dauter Z, Kuznetsov SR, Jones TL, Derewenda ZS (2000). Crystal structure of the human acyl protein thioesterase I from a single X-ray data set to 1.5 A. Structure Fold Des.

[B24] Altschul SF, Gish W, Miller W, Myers EW, Lipman DJ (1990). Basic local alignment search tool. J Mol Biol.

[B25] Marchler-Bauer A, Anderson JB, DeWeese-Scott C, Fedorova ND, Geer LY, He S, Hurwitz DI, Jackson JD, Jacobs AR, Lanczycki CJ, Liebert CA, Liu C, Madej T, Marchler GH, Mazumder R, Nikolskaya AN, Panchenko AR, Rao BS, Shoemaker BA, Simonyan V, Song JS, Thiessen PA, Vasudevan S, Wang Y, Yamashita RA, Yin JJ, Bryant SH (2003). CDD: a curated Entrez database of conserved domain alignments. Nucleic Acids Res.

[B26] Apweiler R, Attwood TK, Bairoch A, Bateman A, Birney E, Biswas M, Bucher P, Cerutti L, Corpet F, Croning MD, Durbin R, Falquet L, Fleischmann W, Gouzy J, Hermjakob H, Hulo N, Jonassen I, Kahn D, Kanapin A, Karavidopoulou Y, Lopez R, Marx B, Mulder NJ, Oinn TM, Pagni M, Servant F, Sigrist CJ, Zdobnov EM (2001). The InterPro database, an integrated documentation resource for protein families, domains and functional sites. Nucleic Acids Res.

[B27] Tatusov RL, Fedorova ND, Jackson JD, Jacobs AR, Kiryutin B, Koonin EV, Krylov DM, Mazumder R, Mekhedov SL, Nikolskaya AN, Rao BS, Smirnov S, Sverdlov AV, Vasudevan S, Wolf YI, Yin JJ, Natale DA (2003). The COG database: an updated version includes eukaryotes. BMC Bioinformatics.

[B28] Leipe DD, Koonin EV, Aravind L (2003). Evolution and classification of P-loop kinases and related proteins. J Mol Biol.

[B29] Walker JE, Saraste M, Runswick MJ, Gay NJ (1982). Distantly related sequences in the alpha- and beta-subunits of ATP synthase, myosin, kinases and other ATP-requiring enzymes and a common nucleotide binding fold. Embo J.

[B30] Aravind L, Iyer LM, Leipe DD, Koonin EV (2004). A novel family of P-loop NTPases with an unusual phyletic distribution and transmembrane segments inserted within the NTPase domain. Genome Biol.

[B31] Saraste M, Sibbald PR, Wittinghofer A (1990). The P-loop--a common motif in ATP- and GTP-binding proteins. Trends Biochem Sci.

[B32] Vetter IR, Wittinghofer A (1999). Nucleoside triphosphate-binding proteins: different scaffolds to achieve phosphoryl transfer. Q Rev Biophys.

[B33] Bernstein NK, Williams RS, Rakovszky ML, Cui D, Green R, Karimi-Busheri F, Mani RS, Galicia S, Koch CA, Cass CE, Durocher D, Weinfeld M, Glover JN (2005). The molecular architecture of the mammalian DNA repair enzyme, polynucleotide kinase. Mol Cell.

[B34] Bairoch A (2000). The ENZYME database in 2000. Nucleic Acids Res.

[B35] Mochizuki K, Oguro A, Ohtsu T, Sonenberg N, Nakamura Y (2005). High affinity RNA for mammalian initiation factor 4E interferes with mRNA-cap binding and inhibits translation. Rna.

[B36] Joshi B, Cameron A, Jagus R (2004). Characterization of mammalian eIF4E-family members. Eur J Biochem.

[B37] Marcotrigiano J, Gingras AC, Sonenberg N, Burley SK (1997). Cocrystal structure of the messenger RNA 5' cap-binding protein (eIF4E) bound to 7-methyl-GDP. Cell.

[B38] Matsuo H, Li H, McGuire AM, Fletcher CM, Gingras AC, Sonenberg N, Wagner G (1997). Structure of translation factor eIF4E bound to m7GDP and interaction with 4E-binding protein. Nat Struct Biol.

[B39] Ishida T, Katsuta M, Inoue M, Yamagata Y, Tomita K (1983). The stacking interactions in 7-methylguanine-tryptophan systems, a model study for the interaction between the 'cap' structure of mRNA and its binding protein. Biochem Biophys Res Commun.

[B40] Morino S, Hazama H, Ozaki M, Teraoka Y, Shibata S, Doi M, Ueda H, Ishida T, Uesugi S (1996). Analysis of the mRNA cap-binding ability of human eukaryotic initiation factor-4E by use of recombinant wild-type and mutant forms. Eur J Biochem.

[B41] Altmann M, Edery I, Trachsel H, Sonenberg N (1988). Site-directed mutagenesis of the tryptophan residues in yeast eukaryotic initiation factor 4E. Effects on cap binding activity. J Biol Chem.

[B42] Vasilescu S, Ptushkina M, Linz B, Muller PP, McCarthy JE (1996). Mutants of eukaryotic initiation factor eIF-4E with altered mRNA cap binding specificity reprogram mRNA selection by ribosomes in Saccharomyces cerevisiae. J Biol Chem.

[B43] Ptushkina M, von der Haar T, Karim MM, Hughes JM, McCarthy JE (1999). Repressor binding to a dorsal regulatory site traps human eIF4E in a high cap-affinity state. Embo J.

[B44] Pyronnet S, Imataka H, Gingras AC, Fukunaga R, Hunter T, Sonenberg N (1999). Human eukaryotic translation initiation factor 4G (eIF4G) recruits mnk1 to phosphorylate eIF4E. Embo J.

[B45] Pons T, Gomez R, Chinea G, Valencia A (2003). Beta-propellers: associated functions and their role in human diseases. Curr Med Chem.

[B46] Anantharaman V, Koonin EV, Aravind L (2001). Regulatory potential, phyletic distribution and evolution of ancient, intracellular small-molecule-binding domains. J Mol Biol.

[B47] Breitbart M, Salamon P, Andresen B, Mahaffy JM, Segall AM, Mead D, Azam F, Rohwer F (2002). Genomic analysis of uncultured marine viral communities. Proc Natl Acad Sci U S A.

[B48] Venter JC, Remington K, Heidelberg JF, Halpern AL, Rusch D, Eisen JA, Wu D, Paulsen I, Nelson KE, Nelson W, Fouts DE, Levy S, Knap AH, Lomas MW, Nealson K, White O, Peterson J, Hoffman J, Parsons R, Baden-Tillson H, Pfannkoch C, Rogers YH, Smith HO (2004). Environmental genome shotgun sequencing of the Sargasso Sea. Science.

[B49] Saini HK, Fischer D (2005). Meta-DP: domain prediction meta-server. Bioinformatics.

[B50] Fischer D, Eisenberg D (1996). Protein fold recognition using sequence-derived predictions. Protein Sci.

[B51] Fischer D (2003). 3D-SHOTGUN: a novel, cooperative, fold-recognition meta-predictor. Proteins.

[B52] FRalanyzer. http://fralanyzer.cse.buffalo.edu.

[B53] Petrey D, Xiang Z, Tang CL, Xie L, Gimpelev M, Mitros T, Soto CS, Goldsmith-Fischman S, Kernytsky A, Schlessinger A, Koh IY, Alexov E, Honig B (2003). Using multiple structure alignments, fast model building, and energetic analysis in fold recognition and homology modeling. Proteins.

[B54] Kraulis PJ (1991). MOLSCRIPT: a program to produce both detailed and schematic plots of protein structures.. J Appl Cryst.

[B55] Jaroszewski L, Rychlewski L, Li Z, Li W, Godzik A (2005). FFAS03: a server for profile--profile sequence alignments. Nucleic Acids Res.

